# Multisensory perception reflects individual differences in processing temporal correlations

**DOI:** 10.1038/s41598-018-32673-y

**Published:** 2018-09-27

**Authors:** Aaron R. Nidiffer, Adele Diederich, Ramnarayan Ramachandran, Mark T. Wallace

**Affiliations:** 1 Department of Hearing and Speech Sciences, Vanderbilt University, Nashville, TN USA; 2 Department of Health, Life Sciences & Chemistry Jacobs University, Bremen, Germany; 3 Vanderbilt Brain Institute, Vanderbilt University, Nashville, TN USA; 4 Department of Psychology, Vanderbilt University, Nashville, TN USA; 5 Department of Psychiatry, Vanderbilt University, Nashville, TN USA; 6 Vanderbilt Kennedy Center, Vanderbilt University, Nashville, TN USA

## Abstract

Sensory signals originating from a single event, such as audiovisual speech, are temporally correlated. Correlated signals are known to facilitate multisensory integration and binding. We sought to further elucidate the nature of this relationship, hypothesizing that multisensory perception will vary with the strength of audiovisual correlation. Human participants detected near-threshold amplitude modulations in auditory and/or visual stimuli. During audiovisual trials, the frequency and phase of auditory modulations were varied, producing signals with a range of correlations. After accounting for individual differences which likely reflect relative unisensory temporal characteristics in participants, we found that multisensory perception varied linearly with strength of correlation. Diffusion modelling confirmed this and revealed that stimulus correlation is supplied to the decisional system as sensory evidence. These data implicate correlation as an important cue in audiovisual feature integration and binding and suggest correlational strength as an important factor for flexibility in these processes.

## Introduction

Our environment provides us with an enormous amount of information that is encoded by multiple sensory modalities. One of the fundamental tasks of the brain is to construct an accurate and unified representation of our environment from this rich array of sensory signals. To accomplish this, the brain must decide which signals arise from a common source. For example, during conversation among a group of individuals, listeners can group appropriate words from the same voice and further associate voices with the appropriate speakers, a process greatly facilitated by the availability of both audible and visible cues^1^. Benefits that are associated with the presence of multisensory signals include increased detection^2^ and localization accuracy^3^, improved speech intelligibility^4^ and speeding of reaction times^5,6^.

A number of principles have been proposed that relate the spatial and temporal proximity of multisensory signals and the manner in which these enhance neural and behavioral responses^2,7–9^. These factors have also been related to our brain’s determination that multisensory signals come from the same source^10,11^. In addition to these principles, it has been demonstrated that the temporal similarity (i.e., correlation) of these signals are also important in shaping our multisensory perception and causal inference^12–15^. Indeed, temporal similarity is a hallmark feature of signals originating from the same source, such as the voice and mouth movements of a speaker^16^, and has been shown to be a robust cue for the binding of unisensory^17,18^ and multisensory^19–22^ features. Observers can utilize these temporal correlations in multisensory signals to enhance behavioral performance^22–24^.

Although we know that temporal correlation between unisensory signals leads to a unified multisensory percept and enhancement of multisensory behaviors, it is not known whether, and if so how, multisensory behavioral performance varies with the strength of the correlation. We hypothesize that audiovisual temporal correlation provides sensory evidence for multisensory decisions that is proportional to the sign and magnitude of the correlation. Further we hypothesize that these graded changes in sensory evidence will result in corresponding changes in multisensory behavior. To test these hypotheses, we presented participants with audiovisual signals with barely detectable (i.e., near threshold) amplitude modulation (AM). While manipulating the temporal correlation between the auditory and visual signals, we measured how observers’ ability to detect these fluctuations changed with changes in stimulus correlation. We propose a mechanism—analogous to a phase shift—that approximates relative differences in unisensory temporal processing and that accounts for individual differences in behavioral results. Finally, we employed drift-diffusion modelling to test whether multisensory behavioral performance is better approximated by absolute stimulus correlation or by the adjusted correlations that account for this phase shift.

## Results

**Figure 1:**
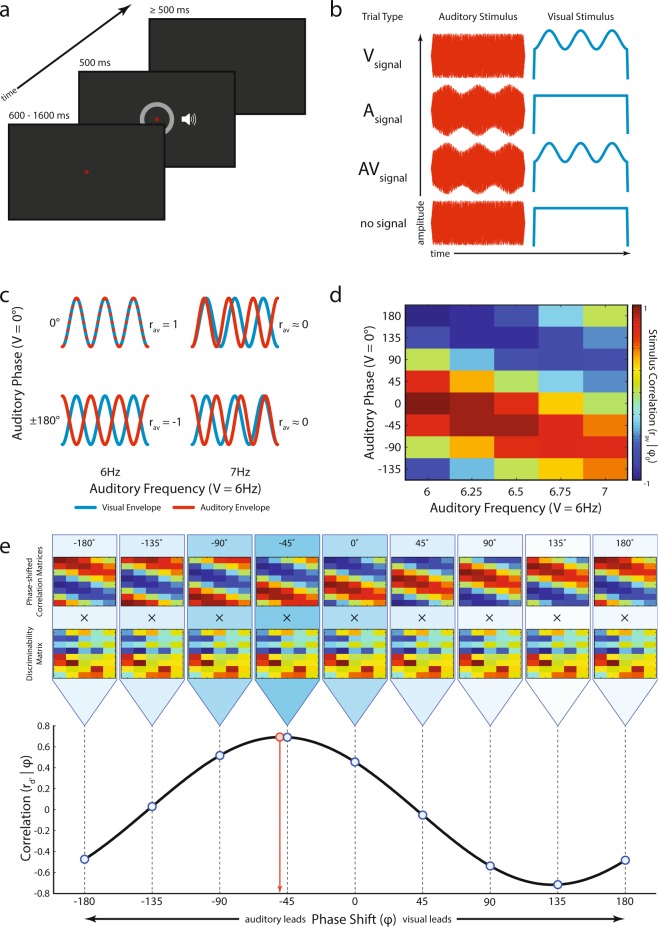
Amplitude modulation detection task. (**a**) Schematic representation of a single trial. Each trial began with the illumination of a fixation target. After a variable wait period, simultaneously presented auditory and visual stimuli appeared (see **b**). Participants indicated the presence or absence of amplitude modulation with a button press. (**b**) Auditory and visual stimuli were always present, but modulation was presented in auditory stimuli only (A_signal_ trial), visual stimuli only (V_signal_ trial), audiovisual stimuli (AV_signal_ trial), or neither stimulus (no signal, catch trial). (**c**) During audiovisual presentations, the frequency and phase of auditory modulation could be independently manipulated yielding a range of audiovisual correlations (r_av_). Correlations were computed using the time series of the auditory and visual envelopes. Note that the visual envelope is always constant while the auditory envelope is varied. Four conditions out of forty are shown for illustration. (**d**) Stimulus Correlation Matrix (r_av_|φ_0_). All forty AV stimulus conditions are shown organized according to Δ frequency × Δ phase. Colors represent the correlation values of audiovisual stimuli across the different frequencies and phases presented where each color box represents one condition. In the task structure, there were 21 unique audiovisual stimulus correlations. (**e**) In order to account for phase shifts in individual participant data, the values in the stimulus correlation matrix (r_av_|φ_0_) were correlated to each participant’s discriminability matrix (r_d’_). In the top panel, a series of correlation matrices are shown in which a phase lag, φ_i_, was applied to auditory (positive shifts) or visual (negative shifts) before correlations were computed, (r_av_|φ_i_). A total of 360 correlation matrices were correlated with the participant’s discriminability matrix (r_d’_; middle panel, nine examples shown), approximating a cross-correlation. In the bottom panel, each of the 360 correlations (r_d’_) was plotted against phase lag [(r_d’_|φ); black line, examples shown by blue dots]. This function was fit to a sine wave and the phase of that fit was extracted (φ’; red dot and arrow) and was taken to represent a participant’s individual phase shift. The stimulus correlations at that phase shift (r_av_|φ’) was taken to represent a participant’s “internal” correlation matrix.

Participants (n = 12) detected near-threshold amplitude modulated (AM) audiovisual stimuli (Fig. 1a,b). The temporal correlation of the AM signals was manipulated by systematic changes in the phase and frequency relationship of the auditory and visual pairs (Fig. 1c). Our central hypothesis was that multisensory behavioral performance would improve commensurate with increasing temporal similarity between the paired audiovisual stimuli (i.e., as correlation become more positive). To examine the potential dependence of behavior on stimulus correlation, a discriminability (*d’*) matrix and a reaction time (RT) matrix for each participant was constructed and related to the stimulus correlation (r_av_) matrix (Δ frequency × Δ phase; Fig. 1d).

**Figure 2:**
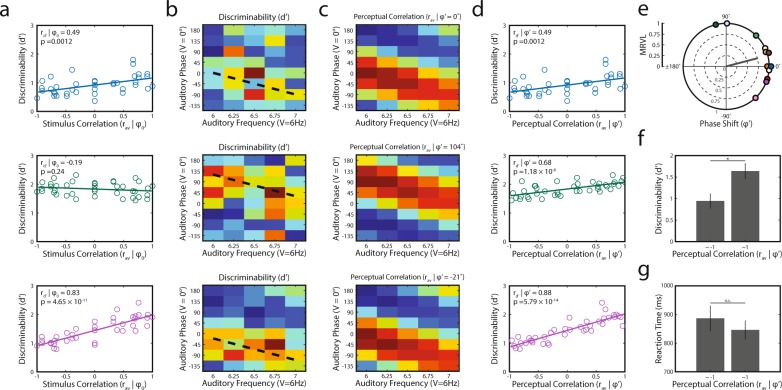
Individual participant data examples. (**a**) Behavioral dependence on stimulus correlation (r_d’_|φ_0_) of three example participants. For parts a-d, each row represents a single participant. Participants are represented by the same color across the figures. (**b**) Discriminability matrices from three participants show how changes in phase (Δ phase; y-axis) and frequency (Δ frequency; x-axis) impact the ability to detect amplitude modulation (discriminability). Diagonal dashed lines represent the computed individual phase shifts (φ’ = 0°, −104°, and 21°) corresponding to the approximate middle of the diagonal of positive correlations in (c). Color values have been scaled separately and range from the lowest to highest value (shown in panel a) for each participant. (**c**) Phase shifted (“perceived”) correlation matrices (r_av_|φ’) from each participant shown in (**a**). Note the strong positive (upward) shift in the second example participant and the moderate negative (downward) shift in the third example participant, relative to Fig. 1d. (**d**) Behavioral dependence (r_d’_|φ’) on perceived stimulus correlation. Each participant shows a strong positive relationship between perceived stimulus correlation and detection behavior (i.e., discriminability). Note that the data in the middle panel was not significantly correlated to physical stimulus correlation (a) but reached significance when accounting for the phase shift. Further, note that the top participant shown did not differ between the two measures due to the lack of observed phase shift. Colors follow the convention described in (**a**). (**e**) Distribution of observed phase shifts from all participants and mean resultant vector. Phase shifts were concentrated around the mean (14.7°, not uniform across phase). Phases were shifted toward positive values (visual leading) but were not significantly different from zero. (**f**,**g**) Accuracy and reaction time effects between stimuli with the strongest negative and positive perceptual correlations. Strong positive correlation improves detection performance but has no impact on reaction times.

While RTs did not show a robust systematic pattern (likely a result of the near-threshold nature of the stimuli, although see Table 1 for RT correlations in some participants), discriminability had a discernible pattern that reflected the nature of the stimulus correlations. In eight of 12 participants, discriminability was significantly correlated with stimulus correlation (Fig. 2a). However, upon visual inspection, the discriminability matrices of two of the remaining four participants mirrored the stimulus correlation matrix but with an apparent shift along the Δ phase dimension (see Fig. 2a,b, middle panels for one example). In fact, this phase shift appeared to be present in most participants to varying degrees and seemed to occur evenly across Δ frequency for each participant (i.e., any shift along the phase dimension was present for all auditory frequencies presented). We therefore hypothesized that this phase shift reflects an internal transformation that alters the relationship between stimulus correlation and behavior (and that is likely driven by individual differences in unisensory temporal processing).

**Table 1:** Reaction time (RT), hit rate (HR) and discriminability (d’) correlations.

Ptc.	RT		HR		d’	
	R	p	R	p	R	p
1	**−0.24**	**0.14**	0.77	4.5e-9	0.76	1.2e-8
2	−0.59	5.3e-5	0.91	4.0e-16	0.91	1.1e-15
3	−0.34	0.031	0.50	0.001	0.49	0.001
4	**0.05**	**0.78**	0.68	8.7e-7	0.68	1.2e-6
**5**	**−0.25**	**0.14**	**0.14**	**0.36**	**0.12**	**0.45**
6	−0.44	0.0042	0.70	4.3e-7	0.68	1.2e-06
7	**−0.21**	**0.19**	0.71	2.7e-7	0.70	4.7e-7
8	−0.54	3.3e-4	0.89	1.2e-14	0.88	5.8e-14
9	**0.05**	**0.75**	0.87	3.1e-13	0.86	8.0e-13
10	−0.39	0.014	0.57	1.3e-4	0.57	1.4e-4
11	−0.41	0.01	0.70	4.9e-7	0.62	2.2e-5
**12**	**0.08**	**0.65**	**0.19**	**0.22**	**0.20**	**0.21**

Nonsignificant correlations are in bold.

### Individuals display unique characteristics for auditory and visual temporal processing

We sought to measure and account for these individualized phase shifts. We modeled this by applying a phase shift to every condition in one of the unisensory modalities before recalculating a stimulus correlation matrix. We then measured the correlation between the discriminability matrix and a series of stimulus correlation matrices computed with phase shifts ranging from −180° to +180° (Fig. 1e; more detail in methods). We then fit this series of correlations to a sine wave. Due to the cyclical nature of the stimulus correlation matrix along the Δ phase dimension, we expected the correlations to be in the shape of a sine wave. As expected, each participant’s phase-shifted correlations were well fit (r^2^ = 0.99999 ± 2.9 × 10^−5^). Another expectation is for these functions to have a period of 360° and to be centered about zero. Indeed, we found no evidence that their period was different from 360° (period = 360.06 ± 0.71; *t*_11_ = 0.2702, *p* = 0.79) or that their center was different from 0 (center = 1.3 × 10^−4^ ± 5.4 × 10^−4^; *t*_11_ = 0.783, p = 0.45). Therefore, we calculated a participant’s phase shift from these fits and then recomputed a unique correlation matrix for each participant using their individual phase shift.

As a test of the validity of phase shift, the pattern of data in the discriminability matrix should mirror the pattern of the phase-shifted stimulus matrix. This would manifest in several ways. First, if the perceived correlation matrix accounts for the data, large changes in the data should be accounted for by changes in the correlations. Therefore, the residual errors between the two measures should be very small relative to the data and centered on zero. Discriminability values (Fig. 2b) were significantly above zero (*d’* = 1.30 ± 0.66; *z* = 43.579, *p* = 8.75 × 10^−169^). Subtracting the predicted *d’*, which was computed from the perceptual correlation matrices (see Methods; Fig. 2c), from the observed *d’*, yielded residual errors which were substantially smaller and less variable compared to *d’* (mean error = 0.018 ± 0.33). Indeed, these residual errors did not differ significantly from zero (*z* = 1.210, *p* = 0.23). Second, we might question the validity of these phase shifts if the data do not mirror perceptual correlations equally for each Δ frequency (e.g., if the diagonal of high *d’* values in the discriminability matrix has a slope that doesn’t match the slope of high *d’* values in the predicted discriminability matrix). To quantify this, we examined residual errors across different frequencies for any systematic changes. Residual error magnitude and variability showed no linear relationships across Δ frequency in any participant (magnitude: slopes = 0.047 ± 0.10, all *p* > 0.12; variability: slopes = 0.016 ± 0.07, all *p* > 0.09). Thus, phase shifts appear to be valid and systematic shifts in the phase dimension are independent of frequency. As such, the correlation matrices constructed using each participant’s unique phase shift could be envisioned to represent the internal (“perceived”) correlations of the external stimuli, accounting for differences in latency of sensory processing between the auditory and visual systems.

These perceptual correlations were used when determining the relationship between discriminability and stimulus correlation (r_d’_; Fig. 2d). The sine wave fits between phase shift and correlation revealed the degree of participant audiovisual phase shift (φ’; Fig. 2e). Phase shifts were not significantly different from 0 across participants but favored a visual leading shift (mean φ’ = 14.7 ± 39.7°; 95% CI [42.2° −12.9°]). The distribution of shifts was concentrated about the mean as indexed by the mean resultant vector length (Fig. 2e; MRVL = 0.76; z = 11.998, p = 1.5 × 10^−8^, Rayleigh Test). To further probe the validity of these phase shifts, we tested whether the magnitude of phase shift was correlated to the strength of the relationship between behavior and stimulus correlation. Smaller correlations associated with larger phase shifts might suggest that the repeated phase shift approach returned spurious correlations. We found no evidence of such a relationship (rho = 0.25, p = 0.68).

### Amplitude modulation discriminability varies with perceived stimulus correlation

Previously, it has been shown that strongly correlated multisensory stimuli provided behavioral and perceptual benefits relative to unisensory performance whereas poorly correlated stimuli fail to provide such benefits^12,20,22^. To examine whether a similar relationship is evident for the current task, we compared the discriminability of stimuli that had the highest and lowest correlation for each participant. We found that discriminability of audiovisual signals with the strongest correlations was better than for audiovisual signals with the strongest anti-correlations (Fig. 2f; t_11_ = 4.312, p = 0.0062, corrected). In contrast, reaction times failed to differ between correlated signals and uncorrelated signals (Fig. 2g; t_11_ = 3.384, p = 0.19, corrected).

**Figure 3:**
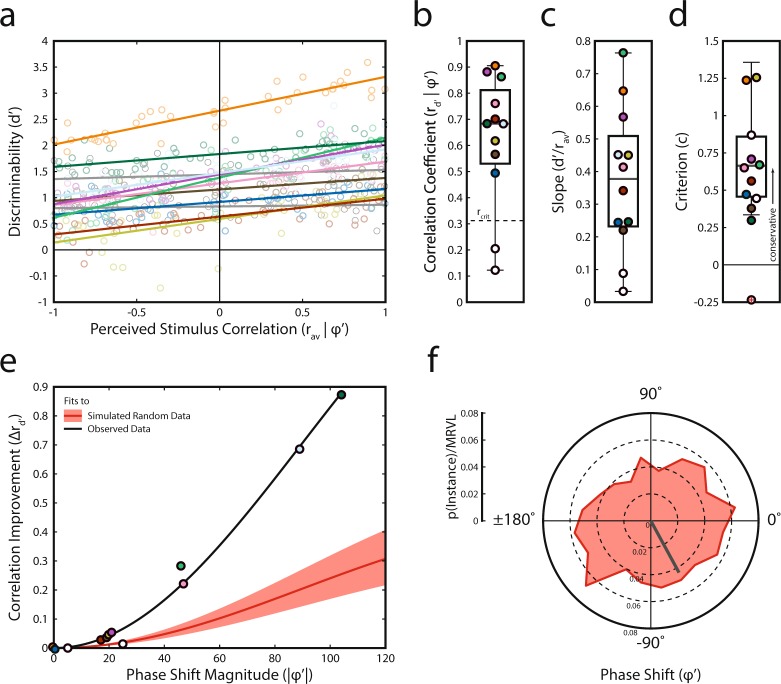
Behavioral results. (**a**) Behavioral dependence on perceived stimulus correlation across all participants (same as Fig. 2d). Behavioral performance in 10 of 12 participants was driven by stimulus correlation. Non-significantly correlated data are represented in grey. Significantly correlated data is depicted in color. As in Fig. 2, each participant retains the same color across the figures. (**b**) Correlation coefficients (r_d’_|φ’) for each participant. The critical value of the correlation coefficient is denoted by a dashed line. (**c**) Slope of linear data fits shown in (**a**) for each participant. (**d**) Criterion for each participant. Each participant but one held a conservative criterion indicating that participants weren’t biased toward responding “yes.” (**e**) Improvement in correlation (Δr_d’_) is associated with phase shift and the effect is larger than expected by chance. Red line and shaded region represent the average fit and 95% confidence bands of random data from the Monte Carlo simulation fit to a sine wave. The black line represents the fit of the observed data to the sine wave. The amplitude of the data fit sine wave was significantly larger than expected by chance. (**f**) Distribution of phase shifts and the corresponding MRVL obtained from the Monte Carlo simulation. In contrast to observed data shown in Fig. 2e, these phase shifts are not significantly concentrated about the circular mean. Note the scale difference in the radial axis between Fig. 2e and here.

Our focus of the current study was to show that multisensory behavior varied proportionally with stimulus correlation. Although we demonstrated above that this relationship was robust in most participants (Fig. 2a), there was evidence that this effect was weakened—and in some participants absent—due to significant individual variability. Thus, it still remained unclear whether phase shift plays an important role in this relationship. To test this, we measured the association between perceived stimulus correlation and discriminability (r_d’_|φ’; Fig. 3a). These correlations were significant in ten out of the twelve participants—two participants more than when not accounting for phase shift. This proportion, 10/12, was significantly greater than expected based on random effects (*p* = 0.019, binomial test). The significant correlations revealed effects that were very strong (Fig. 3b). The correlation values for discriminability and hit rate are presented for each participant in Table 1.

Because we varied auditory parameters while holding visual parameters stationary, it remained possible that participant performance was driven by cues in the auditory modality rather than by audiovisual correlation. In order to rule out that the effects reported here may be a result of unisensory auditory performance, four participants returned and completed a new experiment where visual modulation depth was set to zero while auditory depth was set at their individual threshold. We correlated auditory performance with AM frequency, AM phase, and perceived stimulus correlation. These data are summarized in Table 2. None of these correlations were significant in any of the four participants, even when computing perceived correlations based on potential phase shifts in auditory or audiovisual performance data. Moreover, phase shifts obtained from the auditory data were very different than those obtained from audiovisual data. As a final check, we subtracted the auditory data from the audiovisual data and measured the phase shift and resultant correlation. All four participants showed a significant correlation and the obtained phase shifts corresponded well to the phase shifts obtained from audiovisual data. These results suggest that audiovisual correlations—rather than auditory modulations—are responsible for the behavioral effects presented here.

**Table 2:** Results of auditory only experiments.

Ptc.		Stimulus Correlation Effect on				Frequency Effect on A	Phase Effect on A
		AV	A^a^	A^b^	AV-A		
1	Shift	47	**99**	—	35	—	—
	R	0.76	**0.29**	**0.09**	0.55	**−0.01**	**0.20**
	p	1.2e-8	**0.067**	**0.59**	2.7e-4	**0.93**	**0.43**
6	Shift	−89	**100**	—	−89	—	—
	R	0.68	**0.2**	**−0.18**	0.6	**−0.04**	**0.26**
	p	1.2e-6	**0.21**	**0.22**	4.5e-5	**0.83**	**0.25**
8	Shift	21	**−152**	—	23	—	—
	R	0.88	**0.27**	**−0.27**	0.81	**0.03**	**0.31**
	p	5.8e-14	**0.097**	**0.091**	1.9e-10	**0.85**	**0.14**
10	Shift	−19	**−18**	—	−17	—	—
	R	0.57	**0.05**	**0.04**	0.35	**−0.15**	**0.11**
	p	1.4e-4	**0.76**	**0.82**	0.026	**0.35**	**0.76**

^a^Correlations were unconstraint and reflect best possible correlations. ^b^Correlations were constrained by audiovisual phase shift. Nonsignificant correlations (Fig. 3b) are in bold.

When accounting for phase shift, the strength of these behavioral effects increased in all participants (Δr_d’_ = 0.19 ± 0.29) and the increase was more pronounced in participants with larger magnitude phase shifts (Fig. 3e, a_obs_ = 0.706). Due to the nature of the phase-shift fitting process, simulated random data (details can be found in methods) produces correlational improvement that peaks at ± 180° (a_null_ = 0.205, 95% CI [0.144 0.271]). Nonetheless, the observed effect was significantly larger than what would be expected by these random effects (z = 15.49, p = 4.3 × 10^−54^). Lastly, in contrast to the concentrated distribution of observed phase shifts (Fig. 2e), the distribution of simulated phase shifts was not significantly different from uniform (Fig. 3f; MRVL = 0.04; z = 2.08, p = 0.125, Rayleigh Test). These findings provide strong support for the notion that phase shift reflects an important transformation between stimulus correlation as it occurs in the environment and how it manifests in perceptual performance.

Individuals showed widely varying dependencies on stimulus correlation as measured by the slope of a linear psychometric function fit to discriminability data (Fig. 3c; sig. slopes = 0.43 ± 0.18). Lastly, despite the stimuli being presented at threshold levels, we were concerned about the possibility of participants adopting a strategy that exploits the low proportion of catch trials (i.e., they could be always reporting the presence of the stimulus modulation). We therefore quantified participant’s willingness to respond with “modulation present”. Figure 3d confirms that this strategy was not employed (c = 0.61 ± 0.41) with 11 of 12 participants adopting a conservative criterion. Further reinforcing this, 10 out of 12 participants (including the lone participant with a liberal criterion) were within one standard deviation of an unbiased criterion (−1 < c < 1).

### Perceived stimulus correlation predicts audiovisual behavior via changes in evidence accumulation

Next, we sought to describe how audiovisual temporal correlation and phase shift influence behavioral performance in a decisional framework. Typically, changes in choice frequency and reaction time in a decision task are driven by changes in sensory evidence. We hypothesized that, in our task, sensory evidence was conferred by the temporal correlation of the stimuli. Further, we asked whether perceptual correlations rather than physical correlations better account for changes in behavioral performance on a participant-by-participant basis. To answer these questions, we employed two decision models.

**Figure 4:**
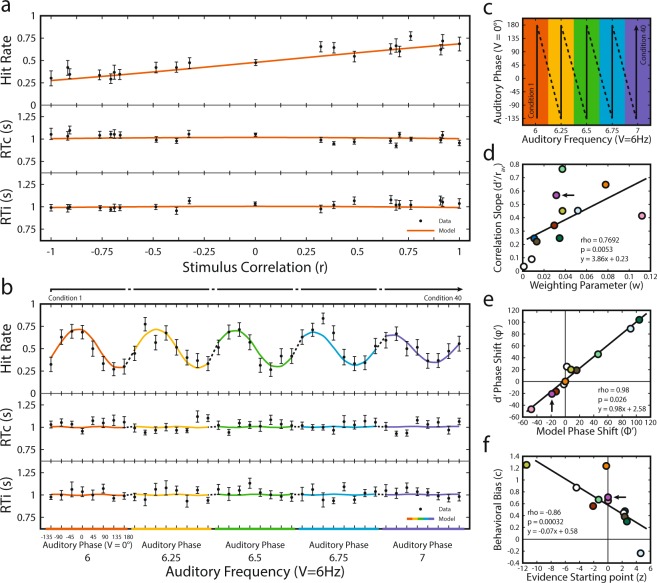
Modeling results and comparison to behavioral results. (**a**) Model 1 fit for a single participant. Proportion of correct responses (top panel), reaction times for correct responses (RTc; middle panel), and reaction times for incorrect responses (RTi; bottom panel) are shown (black dots, ±1 S.E.M) for the 21 unique audiovisual correlations. The same data are shown from the model prediction (red lines). (**b**) Model 2 fit for the same participant. Proportion of correct responses (top panel), reaction times for correct responses (RTc; middle panel), and reaction times for incorrect responses (RTi; bottom panel) are shown (black dots, ± 1 S.E.M) for all 40 audiovisual conditions (top arrow). Model predictions and observed data are shown along a single continuous axis for simplicity with non-continuous data points connected by dashed lines (see panel c for key). (**c**) Representation of experimental conditions (frequency and phase) and how they are represented in panel (b). Conditions are organized in matrices (as in Fig. 2b,c) with columns representing different frequencies and rows representing different phases. In (**b**), data have been reorganized column-wise such that Condition 1 is the first phase in the first frequency and Condition 40 is the last phase in the last frequencies. Colors of the model fit and bottom axis in (**b**) correspond to columns in the matrix with the same color. The top arrow in (**b**) correspond to the arrow in (**c**), unfolded. (**d**) Across all participants, phase shifts measured from discriminability matrices (Fig. 2e) are strongly correlated with the phase shift parameters output by the diffusion model. Participant data shown in (**a**&**b**) correspond to the marker indicated by the arrow. (**e**) Measures of bias, criteria (from Fig. 3d) and evidence starting point parameters are correlated across participants. Participant data shown in (**a**&**b**) correspond to the marker indicated by the arrow. (**f**) Measures of dependence on stimulus correlation, psychometric slopes (from Fig. 3c) and scaling parameters are correlated across participants. Participant data shown in (**a**&**b**) correspond to the marker indicated by the arrow.

The first model assumed that the drift rates, which index sensory evidence, are related to physical stimulus correlations (r_av_|ϕ_0_) across conditions (Fig. 4a). For the second model we assumed that the drift rates are related to the perceived stimulus correlations (Fig. 4b), that is, correlations determined after a phase shift was applied (r_av_|ϕ_i_). This design allowed the models not only to predict choice and reaction times with sensory evidence based on stimulus correlation, but also to measure participant phase shifts, providing converging evidence (in conjunction with results provided above) of an internal phase shift of the representation of the physical stimuli.

Tables 3 and 4 show the estimated parameters for each model and their goodness of fit. Both models were well fit to the data and model 2 successfully incorporated the extra parameter for phase shift without compensation from other parameters meant to index bias, speed/accuracy trade-off and sensory encoding/preparation. As evidence that the models were not simply adjusting other parameters to adjust between models, we found that these parameters were strongly correlated between models when accounting for phase shift using partial correlations (θ: rho = 0.78, p = 0.0046; β: rho = 0.98, p = 7.67 × 10^−8^; T_r_: rho = 0.87, p = 0.00044). Using Akaike Information Criterion (AIC) as a model selection metric, we found that most (8/12) participants’ behavior was better described by the second model, in which the perceived correlation, included as a phase shift parameter, drives the decision process. Qualitatively, perceptual choice across conditions can be described as a dampening oscillator with dampening increasing with Δ frequency, a pattern which is also apparent in the model prediction of choice. Figure 4b shows the model fit (colored lines matching conditions shown in Fig. 4c) to a single participant’s data (filled circles).

**Table 3:** Model 1 parameters.

Ptc.	θ	β	T_r_	w	Χ^2^	AIC
1	13	0.2151	0.5741	0.0182	97.719	−0.281
2	7	0.4954	0.7915	0.0762	87.192	−10.808
3	19	2.3893	0.6113	0.0111	175.113	77.113
4	9	3.5568	0.7605	0.0001	70.945	−27.055
**5**	**13**	**−4.4554**	**0.8**	**0.0018**	**125.043**	**27.043**
6	9	6.2835	0.6364	0.0001	95.408	−2.592
7	8	−0.6953	0.6018	0.0334	142.682	44.682
8	15	0.1974	0.788	0.0292	108.722	10.722
9	17	0.1539	0.7956	0.025	131.553	33.553
10	5	1.3622	0.7738	0.0339	88.424	−9.576
11	16	−11.924	0.588	0.0333	163.444	65.444
**12**	**9**	**3.2324**	**0.7994**	**0.0089**	**142.744**	**44.744**

Participants (Ptc.) with nonsignificant correlations (Fig. 3b) are in bold. θ = boundary separation, β = evidence starting point, T_r_ = residual time, w = drift-rate scaling parameter.

**Table 4:** Model 2 parameters.

Ptc.	Φ	θ	β	T_r_	w	Χ^2^	AIC
1	46	16	1.3753	0.4859	0.0214	214.55	14.55
2	0	7	−0.2467	0.7881	0.078	158.4	−41.6
3	0	19	2.3988	0.6074	0.011	281.68	81.681
4	−104	6	2.6569	0.7915	0.0348	136.03	−63.97
**5**	**−2**	**13**	**−4.4437**	**0.8**	**0.0012**	**217.53**	**17.528**
6	−92	8	4.6167	0.6443	0.0519	181.62	−18.382
7	13	10	−2.0867	0.5639	0.0298	252.04	52.041
8	19	15	0	0.7863	0.0317	168.74	−31.256
9	−46	17	−1.3041	0.8	0.0374	208.79	8.79
10	−16	14	2.3778	0.6013	0.0133	237.15	37.146
11	−8	15	−11.4521	0.6079	0.0375	225.2	25.201
**12**	**2**	**9**	**2.2282**	**0.7951**	**0.0086**	**242.31**	**42.309**

Participants (Ptc.) with nonsignificant correlations (Fig. 3b) are in bold. Φ = phase shift, θ = boundary separation, β = evidence starting point, T_r_ = residual time, w = drift-rate scaling parameter.

Model 2 made accurate predictions of behavioral choice and reaction times based on the perceptual correlations and returned parameters that closely matched their signal detection theory counterpart. Each participant’s model-fit phase shift parameter (*ϕ’*) nearly perfectly matched their phase shift obtained from discriminability (*φ’*, Fig. 4d; rho = 0.98, p = 0.026, slope = 0.98). Additionally, evidence starting point, *β*, which is the parameter that measures the participant’s bias toward one response over another^25,26^, was also correlated with the signal detection theory measure of bias, *c* (Fig. 4e; rho = 0.77, p = 0.0053). The bias reflects the participant’s tendency to respond with “modulation present” or “modulation absent”, which is unrelated to the sensitivity of the participant. Lastly, the drift-rate weighting coefficient was strongly correlated with the slope of their psychometric functions (Fig. 4f; rho = −0.86, p = 0.00032), with both measures describing the dependence of behavior on changes in correlation.

## Discussion

Temporal factors such as (a)synchrony have long been known to influence multisensory processes in the brain^7,27–31^ and in behavior^5,32–37^. More recently, Parise and colleagues^20^ presented evidence that the fine temporal structure of an audiovisual stimulus *independent of asynchrony* can influence multisensory perception. They further showed that it is possible to explain a number of multisensory phenomena based on a general correlation detection mechanism^24^.The findings presented in the current study provide additional and unique support for the growing evidence implicating temporal correlation as an important cue in multisensory processing.

In the current work we extend this knowledge about multisensory temporal dependencies by showing that audiovisual detection behavior is a monotonic function of stimulus correlation. As the temporal similarity of two unisensory signals increased, detection of amplitude modulation embedded in the audiovisual signal improved in a linear manner (Fig. 3a). Additionally, we qualify this finding in a way that provides mechanistic insight into how the brain combines dynamic stimuli across sensory modalities. Thus, the temporal correlation of the audiovisual stimuli did not necessarily map directly onto multisensory behavioral performance; conditions in which physical stimulus correlation was highest did not always result in the best behavioral performance. Instead, it appears that a transform occurs in the brain of each individual and that results in a phase shift in behavioral performance relative to physical stimulus correlation (r_av_|*φ*_0_). Calculating temporal correlation after applying a phase lag to one of the stimuli (r_av_|*φ’*), which simulates differential processing times for sensory signals in the brain, accounts for this difference. These phase-shifted correlations presumably represent the correlations as they are available to our decisional system.

Although our task did not reveal any measurable effects of temporal correlation on reaction times, we are not surprised. This lack of effect can be explained in terms of RT variability. Our stimuli employed near-threshold signals which are known to produce reaction times that are more variable than those produced by supra-threshold signals^38^. Additionally, the correlations in some stimulus conditions unfolded over time. In contrast, for some conditions the correlation does not change throughout the course of the signals. For example, when the auditory and visual modulations are both at 6 Hz, across the entire stimulus, the relationship is maintained regardless of phase. However, when the frequencies of visual and auditory AM are different (e.g., 6 Hz and 7 Hz, respectively), the starting and ending phase relationships change. In one phase condition (see Fig. 1c), stimuli start out of phase (strong negative correlation) and end in phase (strong positive correlation). In another they start in phase and end out of phase. However, both of these conditions have an averaged correlation of 0 across the entire stimulus duration. This difference could introduce more reaction-time variability in some conditions than others, which may mask potential RT effects in some participants. To better measure any potential effect on reaction times, future experiments should be designed using supra-threshold signals that generate more reliable reaction times and take into account how correlations unfold over time.

The current study strongly grounds the relationship between stimulus correlation and multisensory processing in a decisional framework. Our model successfully incorporated the relationship between two signals (i.e., temporal correlation) into a dynamic-stochastic approach to account for choice frequency and response time. With only very few parameters (4 for model 1 and 5 for model 2) stimulus correlation was able to account for the observed patterns. Moreover, it was able to account for individual differences within and across participants. Our primary finding is related to the nature of how stimulus correlation influences the accumulation of sensory evidence for a decision. Specifically, we found that perceived (phase-shifted) stimulus correlation serves as a good predictor of behavior when used to constrain drift rate. For perceptual tasks, drift rate is often interpreted as an index for the quality (e.g., strength) of sensory evidence that is available to the decisional system^39,40^. Typically, the strength of sensory evidence is provided by the physical attributes of the stimulus, for instance, the degree of motion coherence^41^, intensity^42^, line length^43^, or numerosity^44^. For simple multisensory behaviors (e.g., detection of simple stimuli), the drift rate relates to the combined evidence obtained from integrating the physical stimulus properties across modalities^42,45^, especially when these properties are weak or ambiguous (e.g., low intensity, poor motion coherence, etc.) in the unisensory component stimuli^42^.

In the current task, the key physical parameter that would presumably modulate the magnitude of evidence for detection is the depth of the amplitude modulation, with strength of evidence increasing with depth. However, modulation depth, and thus sensory evidence from the unisensory signals, is held constant across conditions. Although we cannot rule out that evidence is supplied by integration of the unisensory stimulus properties, sensory evidence cannot come from these alone but instead is generated via a computation involving both stimuli. Different types of multisensory decisions require different architectures that depend on the structure of the task or stimulus^46^. The results presented here—that the strength of sensory evidence is based on a computation of the unisensory signals rather than the strength of the unisensory signals themselves—suggests that unisensory signals converge and evidence is computed prior to being evaluated by the decisional system. Other multisensory decisions such as simultaneity judgement^47^ and temporal order judgement^48,49^, which require a similar comparison of the unisensory signals, have also been described in terms of their cross-modal computations.

It has recently been discussed that the presence or absence of audiovisual temporal correlation is a strong determinant of multisensory binding^21^ which manifests in a variety of behavioral enhancements^20,22,23^. Results presented here extend this concept, despite the substantially different nature of the stimuli and task employed. According to our results, multisensory benefits—and likely by extension the propensity to bind two signals—are monotonically related to the strength and sign of the temporal correlation (similarity) between unisensory signals. This notion implies that the process of binding signals is probabilistic. Stochastic binding related to temporal correlation could be an important mechanism in cognitive flexibility. It must be noted that weak, yet often significant, correlations exist in randomly paired stimuli^16^. In a sensory-rich environment, compulsory binding based on temporal similarity could lead to the perceptual unification of unrelated stimuli, creating great ambiguity in deciphering the sensory world. Instead, since the perceptual system has access to the strength of the correlation, the strongest and likely most appropriate signals can be bound. Further, it’s likely that binding and integration are built on several other features such as spatial and temporal proximity. In the natural environment, these features are very often aligned; a single event will produce energies across different modalities that overlap in space and time and that are temporally correlated. Where these features are somewhat discrepant, the brain will appropriately weight (i.e., according to their reliability) proximity and similarity in the construction of a multisensory percept^50,51^.

The perceptual benefits of increased stimulus correlation are likely the result of mechanisms involving synchronized or coherent neural activity across brain regions^52^. Neural coherence has been hypothesized to play a role in shaping our conscious experience^53^ by underpinning mechanisms of sensory awareness^54^, attentional selection^55^, cognitive flexibility^56^, and perceptual binding^17,57–59^. Further, temporally correlated audiovisual streams have been shown to improve the representation of the auditory stimulus envelope and features in auditory cortex^60^. This enhanced representation is likely the end result of why seeing a speaker’s face improves speech intelligibility^4,23,61^. Rhythmic auditory and visual stimuli like the ones used in the current study are known to entrain neural oscillations^52,62,63^ which index patterns of neuronal excitability over time^64^. Since uni- and multisensory stimuli can simultaneously entrain oscillations in multiple frequency bands^52,65^, it is likely that our stimuli do the same and thus induce coherent brain activity commensurate with the correlation in the stimuli.

In the current study, participants’ behavioral performance was not necessarily best for the stimuli with highest physical correlation but were instead phase-shifted by differing amounts for each participant. Behavior very closely matched the correlation of the modulations after a phase lag was applied to one of the modulation signals. This phase lag could be adjusting for different processing times and abilities of participants’ auditory and visual systems. It’s known that oscillations entrain to rhythmic auditory stimuli at different phase lags across listeners^63^. It is possible that visual entrainment occurs in a similar manner and that these phase lags differ between the auditory and visual systems, though we are not aware of such data. Interestingly, phase lag of the entrained oscillations can be calibrated to the particular temporal structure of an audiovisual stimulus^66^. Thus, the phase lags reported in the current study are likely a “preferred” or “natural” phase that can be easily manipulated depending on context (e.g., attending an event that is near or far from the body which would result in different temporal relationships between auditory and visual representations in the brain) in a manner similar to the phenomenon of recalibration of the perception of audiovisual simultaneity^37,67^.

During multisensory decisions, temporal correlation between the features of the component stimuli modulates behavior. It does so by changing the nature of the sensory evidence that is evaluated by the sensory system. The strength of the sensory evidence is proportional to the strength of the correlation of the signal. Finally, the physical correlations present in stimuli are transformed, via a phase shift, into “perceptual” correlations that are unique to an individual. This process likely occurs through differences in unisensory temporal processing. This was confirmed by a dynamic-stochastic model in which the drift rate was related to physical or to perceived correlations between the auditory and visual signals in the audiovisual presentation. These results motivate several fundamental questions. Is binding truly stochastic? Can cross-modal correlation embedded in one feature (e.g., intensity) have the same proportional effect on behavioral performance reported here in tasks utilizing orthogonal stimulus features (e.g., frequency or timbre)? What are the neural signatures of this proportional change and their relation to behavior? Finally, does the perception of naturalistic audiovisual stimuli such as speech benefit in the same way with changes in audiovisual correlation?

## Materials and Methods

### Participants

Twelve individuals (age = 26.4 ± 5.1, seven females) participated in the current study. All participants reported normal or corrected-to-normal vision and normal hearing and were right handed. The study was conducted in accordance with the declaration of Helsinki, and informed written consent was obtained from all participants. All procedures were approved by the Vanderbilt University Institutional Review Board. When applicable, participants were given monetary compensation for participation.

### Apparatus and stimuli

All stimuli were generated in MATLAB (The MathWorks, Inc., Natick, MA) and presented using PsychToolbox version 3^68,69^. Auditory stimuli were digitized at 44.1 kHz, and presented through calibrated open-back circumaural headphones (Sennheisser HD480). Visual stimuli were centered about a red fixation dot in the center of a dark (0.15 cd/m^2^) viewing screen (Samsung Sync Master 2233rz, 120 Hz refresh rate).

Auditory stimuli were frozen tokens of white noise (generated by the *randn* function) at moderate baseline level (48 dB SPL, A-weighted). Visual stimuli consisted of a moderately bright ring (24 cd/m^2^ at baseline; inner diameter: 1.8°, outer diameter: 3.6° visual angle). Both stimuli were presented simultaneously, lasted 500 ms, and were gated by a linear 10 ms onset and offset ramp. Stimulus timing was confirmed with a Hameg 507 oscilloscope, photodiode, and microphone.

For each stimulus, auditory intensity and visual luminance, *y*, could be modulated around their baseline over time, *t*, such that

1 $$y\,(t)=[1+m\,(t)]\times c(t)$$

where

2 $$m\,(t)=M\times sin(2\pi {f}_{m}t+{\phi }_{0,j})$$

and *c(t)* is the time series of the carrier stimulus (auditory: noise; visual: ring). The form of the amplitude modulation (AM) signal *m(t)* is defined by a modulation depth *M* which represents the amplitude of the modulation signal as a proportion of the amplitude of the carrier signal and ranged from 0 (no AM) to 1 (full AM), frequency *f*_*m*_ in Hz, and starting phase *φ*_0*,j*_ in degrees.

On any given trial, the AM signal could be present in the auditory channel alone, the visual channel alone, both channels (audiovisual trials), or neither (catch trials; Fig. 1b). If present, modulation depth was set to individual unisensory thresholds (see below for thresholding procedures). Unisensory signals (AM was present in auditory stimulus only or visual stimulus only) were always presented in cosine phase such that the modulation began at the trough (*φ* = 0°) and at the same frequency (*f*_*m, visual*_ = 6 Hz). When AM was present in both stimuli, visual modulation was always 6 Hz and cosine starting phase while auditory signals could be presented at various frequencies (*f*_*m, auditory*_ = {6, 6.25, 6.5, 6.75, 7 Hz}) and initial phases (*φ*_*0*_ = {−135, −90, −45, 0, 45, 90, 135, 180°}, with *φ*_*0,j*_ *ϵ* φ_0_). This structure results in a total of 40 (5 × 8) different audiovisual stimulus conditions.

Because we are interested in the temporal correlation between the two signals, the Pearson correlation between the auditory and visual envelopes (r_av_) was computed for each of the 40 audiovisual conditions (Fig. 1c). For example, when the auditory and visual envelopes were characterized by the same frequency and phase, correlation was 1. Conversely, stimuli of the same frequency but presented anti-phase resulted in a correlation of −1. The parameters chosen resulted in a representation of correlations between −1 and 1. A stimulus correlation matrix (r_av_|φ_0_) was constructed for all audiovisual conditions by organizing the correlation values according to their frequency and phase relationship between auditory and visual signals (Δ frequency × Δ phase; Fig. 1d).

### Procedure

Participants were seated comfortably inside an unlit WhisperRoom™ (SE 2000 Series) with their forehead placed against a HeadSpot™ (University of Houston Optometry) with the forehead rest locked in place such that a participant’s primary eye position was centered with respect to the fixation point at the center of the viewing screen. Chinrest height and chair height were adjusted to the comfort of the participant.

Prior to the main experiment, each participant completed two separate 3-down 1-up staircase procedures to obtain 79.4% modulation depth thresholds for auditory and visual AM at 6 Hz. For these staircase procedures, on a given trial (Fig. 1a), the red fixation dot appeared at the center of the screen. Participants were instructed to fixate the dot for its entire duration. After a variable time, either an auditory or visual stimulus was presented in which the presence of modulation was determined at random for each trial. Participants were instructed to report the presence of amplitude modulation (described as “flutter”) after the stimulus presentation by pressing “1” on the number pad of a computer keyboard if the modulation was present or pressing “0” if the modulation was absent. The modulation depth decreased after three successive correct responses and increased after one incorrect response. At the beginning of each staircase, the step size was set to increase or decrease modulation depth by 0.05. After two reversals (correct to incorrect response or incorrect to correct response), step size was reduced to 0.025. Finally, after eight reversals, step size became 0.01 in order to arrive at an accurate estimate of modulation depth threshold. Each staircase terminated after 20 reversals. Threshold was determined to be the average of the modulation depth at the last 10 reversals. Instructions included an example of a stimulus with AM at the initial starting modulation depth (*M* = *0.5*) and an example of a stimulus with no AM. So that there was no ambiguity in cases where the first trial did not include a modulation signal, participants were informed that the first trial would have the same modulation depth as the example if present. To control for “runs” of trials with no modulation during the staircase (which could result in erroneously low threshold estimates), a sequence of two trials containing no modulation was always followed by a trial with modulation. The auditory staircase was always completed first and served as a period of dark adaptation prior to the visual staircase.

The main experiment consisted of four blocks lasting approximately 30 minutes each. Each block consisted of 10 trials of each stimulus condition (420 signal trials per block). Additionally, there were catch (no signal) trials included to make up 10% of total trials for that block (47 catch trials per block). Therefore, each block was identical in trial composition (467 total trials per block) but with individual trials presented in a predetermined, pseudorandom order. Each participant completed a total of 1868 trials over the four blocks. Breaks were offered frequently (every 100 trials) to prevent fatigue. Participants completed the full experiment in 2–4 sessions, never completing more than 2 blocks during a session. If a participant completed two blocks in a single session, they were given the opportunity to stretch and walk around while the experimenter set up the second block. Before each block and after any break where the participant was exposed to normal light levels, participants were dark adapted for five minutes. Trials during the main experiment were identical to staircase trials with three exceptions. First, in each trial, both auditory and visual stimuli were presented. Modulation signals could be present in the visual channel alone (V_signal_), auditory channel alone (A_signal_), in both (AV_signal_; with frequency and phase configuration discussed above), or neither channel (no signal). Second, modulation depth was set to a participant’s unique auditory and visual modulation depth thresholds. These threshold values are shown in Table 5. Last, participants were told that they should respond as soon as they had made their decision and were instructed to respond as quickly and accurately as possible. In addition to the participant’s choice, response times were recorded for each trial, sampling every 2.2 μs (4.6 kHz). Response window was terminated after 1.5 seconds. Subsequent responses were censored. This ended up being 2% of trials or less for most participants.

**Table 5:** Participant modulation depth thresholds.

Ptc.	Aud.	Vis.
1	0.041	0.047
2	0.081	0.059
3	0.028	0.049
4	0.104	0.076
5	0.051	0.042
6	0.087	0.062
7	0.068	0.043
8	0.048	0.040
9	0.060	0.058
10	0.063	0.043
11	0.072	0.050
12	0.072	0.070

### Behavioral Analysis

Discriminability (*d’*; a measure of sensitivity) for each of the 40 audiovisual conditions and two unisensory conditions was computed from the relative frequencies of the respective responses,

3 $$d^{\prime} =z({H}_{i})-z(F)$$

where *H*_*i*_ is the proportion of hits (“1”|modulated stimulus) for the i^th^ condition, *F* is the proportion of false alarms (“1”|no modulated stimulus), and *z* is the inverse of the normal distribution function (MATLAB’s *norminv* function) and converts the hit rates and false alarm rates into units of standard deviation of a standard normal distribution. *d’* was organized into a matrix in the same manner as the stimulus correlation matrix. Because the proportion of catch trials was held low and errors had no associated cost^70^, participants could potentially adopt a strategy of simply pressing “1” which would result in a correct choice more often than not. To account for this, criterion (*c*; a measure of bias) for each participant was computed in a similar manner such that

4 $$c=z(H)+z(F)$$

where *H* is the proportion of hits across all conditions. A single criterion was computed for each participant.

To account for individual differences, which became apparent in assessing the phase shift in the *d’* matrices, a series of correlation matrices based on the stimulus correlation matrix (r_av_|φ_0_) were computed after iteratively applying a single degree phase lag to one stimulus (i.e., *φ*_1_ = {−134, −89, −44, 1, 46, 91, 136, −179°}, *φ*_2_ = {−133, −88, −43, 2, 47, 92, 137, −178°}, in general *φ*_*i*_ = {−135 + *i*, −90 + *i*, −45 + *i*, 0 + *i*, 45 + *i*, 90 + *i*, 135 + *i*, 180 + *i*} with *i* = −180, …, 180, resulting in a total of 360 different matrices). A phase-shifted correlation matrix (r_av_|*φ*_*i*_) could be conceptualized as the “internal” or “perceived” correlation of the signals given a particular phase lag, *i*, of one of the signals. Each of the phase-shifted correlation matrices (Fig. 1e, nine examples shown) was in turn evaluated for correlation (r_d’_) with the discriminability matrix of each participant. The resulting correlation values (r_d’_|φ) were then fit to a sine wave using the nonlinear least-squares method. The phase shift value of the fitted sine wave was recorded for each participant (φ’). The CircStat toolbox^71^ was used to describe the nature of the phase shifts and compute the directional statistics across the sample of participants. The “perceptual” correlation matrix corresponding to each participant’s unique phase shift (r_av_|φ’) was used to measure the dependence of behavior on perceived correlation (r_d’_|φ’).

To show that phase shift is related to a central mechanism (e.g., a relative difference in processing latencies between auditory and visual systems), we tested whether the phase shift occurred systematically across all Δ frequencies within each participant. First, a predicted discriminability matrix was calculated from phase-shifted correlations. Phase-shifted correlation matrices were normalized to each participant’s discriminability range by scaling and shifting each unique correlation matrix such that the correlation values at the maximum and minimum correlation matched the *d’* values at the corresponding locations in the discriminability matrix. Next, the values in the predicted discriminability matrix were subtracted from the actual discriminability matrix, resulting in a matrix of residual errors. Then, a linear model was used to determine the relationship (i.e., slope) between Δ frequency and the magnitude and variability (standard deviation) of errors. To calculate significance of variability slope across Δ frequency, a permutation test was used that shuffled the Δ frequency label of errors before calculating standard deviation within each Δ frequency and then fitting a line to the shuffled standard deviations.

We sought to demonstrate that accounting for phase shift improved the measured correlation between behavior and stimulus correlation. Therefore, we computed this dependence on stimulus correlation (r_d’_|φ_0_) and subtracted it from the dependence on perceived correlation discussed above (r_d’_|φ’) which yielded a score of improvement (Δr). Because of the nature of the phase shift fitting process described above, (r_d’_|φ’) ≥ (r_d’_|φ_0_) with the difference growing to a maximum when φ =  ± 180° even for data with no effect (random numbers). Therefore, we accounted for this statistical effect by running a simulation where we computed the phase shift (same process described in Fig. 1e) of 1000 matrices of shuffled data from participants chosen at random. For each matrix, we measured (r_d’_|*φ’*) and (r_d’_|φ_0_) and subtracted them as above so that we had 1000 pairs of φ’ and Δr. These data, along with our observed data, were fit to the function

5 $${\rm{\Delta }}r=a\times sin(\phi ^{\prime} )+a$$

which returned *a*, the amplitude of the function. We then bootstrapped (10000 samples of 20 randomly drawn pairs of simulated φ’ and Δr chosen with replacement) fits to the simulated data to obtain a distribution of *a* for these null data (*a*_*null*_). From this distribution, we computed a z-score for the observed amplitude parameter as

6 $$z=\frac{{a}_{obs}-{a}_{null}}{(u-l)/(2\,\times \,1.96)}$$

where *a*_*obs*_ is the amplitude parameter of the fit to the observed data and *u* and *l* are the upper and lower 95% confidence bounds from the bootstrapped fits to the shuffled data, respectively.

### Diffusion Model Analysis

For binary choices, sequential-sampling models assume that upon presentation of the stimulus, the decision maker sequentially samples information from the stimulus display over time, which provides sensory evidence to a decision process. It also assumes that the decision process accumulates this evidence in a noisy manner for choosing one option over the other, here “modulation present” or “modulation absent.” Sequential-sampling models account simultaneously for choice frequency and choice response times. However, the focus here will be on choice frequencies. Let X(t) denote the random variable representing the numerical value of the accumulated evidence at time *t*. A bias, *β*, (i.e., prior beliefs about the stimulus before it is presented) can influence the initial starting position of the decision process, X(0). This initial state may either favor choice option “modulation present” (X(0) > 0) or choice option “modulation absent” (X(0) < 0). X(0) = 0 reflects an unbiased response. (The initial states can also be given a probability distribution). The participant then samples small increments of evidence at any moment in time, which either favor response “modulation present” (dX(t) > 0) or response “modulation absent” (dX(t) < 0). The evidence is incremented according to a diffusion process. In particular, we apply a Wiener process with drift, lately called drift-diffusion model^72^ with

7 $$dX(t)=\delta +\sigma dW(t)$$

The drift rate, *δ*, describes the expected value of evidence increments per unit time. The diffusion rate, *σ*, in front of the standard Wiener process, *W(t)*, relates to the variance of the increments. Here we set *σ* = 1. The small increments of evidence sampled at any moment in time are such that they either favor response “modulation present” (*dX(t)* > 0) or response “modulation absent” (*dX(t)* < 0). This process continues until the magnitude of the cumulative evidence exceeds a threshold criterion, *θ*. That is, the process stops and response “modulation present” is initiated as soon as the accumulated evidence reaches a criterion value for choosing response “modulation present” (here, *X(t)* = *θ* > 0), or it stops and a “modulation absent” response is initiated as soon as the accumulated evidence reaches a criterion value for choosing response “modulation absent” (here, *X(t)* = *θ* < 0). The probability of choosing the response “modulation present” over “modulation absent” is determined by the accumulation process reaching the threshold for response “modulation present” before reaching the threshold for response “modulation absent”. The criterion is assumed to be set by the decision maker prior to the decision task. The drift rate may be related to the quality of the stimuli (i.e., the better the quality the higher the drift rate). For instance, stimuli that are easier to discriminate are reflected in a higher drift rate. In the following we consider two models. In Model 1 we assume that the physical correlation between the auditory and visual stimuli, (r_av_|φ_0_), weighted by the decision maker drives the evidence accumulation process for initiating a “modulation present” or “modulation absent” response. That is, the drift rate is defined as

8 $$\delta =w\times ({r}_{av}|{\varphi }_{0})$$

Of the 40 correlation coefficients several of them were identical (for instance, a 6 Hz auditory stimulus with starting phases of +45° and −45° both resulted in a correlation of 0.7075) resulting in 21 unique correlation coefficients and by that in 21 different drift rates.

In Model 2 we assume that the physical correlation between the auditory and visual stimuli is distorted by a shift in phase as perceived by the decision maker. That is, the drift rate is defined by

9 $$\delta =w\times ({r}_{av}|{\varphi }_{i})$$

where *i* is a free parameter of the model estimated from the data and its returned value corresponds to a phase shift that is unique to each participant (*ϕ’*). The model term *ϕ*_*i*_ relates to the initial phase term *φ*_*i*_ introduced earlier and follows the same naming conventions. A phase shift unequal to 0, ± 45, ± 90, ± 135, or ± 180 results in 40 different correlation coefficients which in turn results in 40 drift rates.

### Model parameters

We assume for both models that the observed response time is the sum of the decision time, modeled by the diffusion process, and a residual time, *T*_*r*_, which includes the time for processes other than the decision, e.g., sensory encoding and motor components. Here, *T*_*r*_, is a constant for each participant. Because correlation coefficients varied between 1 and −1 but none of the participants showed perfect performances (e.g. 100% of correct responses to either a perfectly positively correlated stimulus pair or a perfectly negatively correlated stimulus pair), we allow an adjustment by including a weight for the correlations 0 ≤ *w* ≤ 1. We also allow for an a priori response bias, *β*, in favor of one response (present/absent). The decision criteria are *θ* = |−*θ*|.

In addition to these parameters, Model 2 returns a parameter *ϕ’* to account for perceived correlations based on individual phase shifts (rather than correlations based on the physical stimuli only) to be estimated from the data. To summarize: For Model 1 four parameters (*w*, *β*, *θ*, *T*_*r*_) are estimated from 63 data points (21 relative frequencies for correct responses, 21 mean response times for correct responses, 21 mean response times for incorrect responses. Trials with identical correlations were collapsed.) For Model 2 five parameters (*ϕ’*, *w*, *β*, *θ*, *T*_*r*_) are estimated from 120 data points (40 relative frequencies for correct responses, 40 mean response times for correct responses, 40 mean response times for incorrect responses).

The model was implemented in terms of the matrix approach^73^ and parameters were estimated by minimizing the chi-square function^74^,

10 $${\chi }^{2}=\sum {(\frac{R{T}_{obs}-R{T}_{pred}}{S{E}_{R{T}_{obs}}})}^{2}+{\sum (\frac{P{r}_{obs}-P{r}_{pred}}{S{E}_{P{r}_{obs}}})}^{2}$$

using the optimization routine *fminsearchbnd* in MATLAB. The *fminsearchbnd* routine is similar to the standard *fminsearch* routine except that the range of the parameters of the parameters can be predetermined, for instance, positive real numbers for the residuals, or real numbers between 0 and 1 for the weights. The *fminsearch* uses the Nelder-Mead simplex search method^75^. $S{E}_{R{T}_{obs}}$ and $S{E}_{P{r}_{obs}}$ refer to the standard error of the observed mean response times and relative choice frequencies, respectively. Note that mean response times and relative choice frequencies are conditioned on the stimulus presented. Here we consider only the trials in which a modulation was present.

For both models, the following procedures/restrictions to parameter values were imposed in the estimation procedure: The decision criteria (absorbing boundaries) were estimated using a search grid. This was done because it quickens the estimation procedure when boundaries are integers (matrix approach). *θ* ranged from 3 to 20 in steps of 1. The residual time, *T*_*r*_, was restricted to 100 ms ≤ *T*_*r*_ ≤ 800 ms and the weight to 0.0001 ≤ *w* ≤ 1. For the Model 2 parameter *ϕ*_*i*_, the value of *i* was restricted to integers ranging from −180 to 180 in steps of 1. For each value of *i* in Model 2, a different set of correlations was computed.

## Data Availability

The datasets generated and analyzed during the current study are available from the corresponding author on reasonable request.
